# Association of Anthropometric Indices With the Development of Diabetes Among Hypertensive Patients in China: A Cohort Study

**DOI:** 10.3389/fendo.2021.736077

**Published:** 2021-10-05

**Authors:** Yingshan Liu, Xiaocong Liu, Shuting Zhang, Qibo Zhu, Xiaoying Fu, Hongmei Chen, Haixia Guan, Yinghua Xia, Qun He, Jian Kuang

**Affiliations:** ^1^ The Second School of Clinical Medicine, Southern Medical University, Guangzhou, China; ^2^ Department of Endocrinology, Guangdong Provincial People’s Hospital, Guangdong Academy of Medical Sciences, Guangzhou, China; ^3^ Department of Cardiology, Guangdong Cardiovascular Institute, Guangdong Provincial People’s Hospital, Guangdong Academy of Medical Sciences, Guangzhou, China; ^4^ Guangdong Provincial Institute of Public Health, Guangdong Provincial Center for Disease Control and Prevention, Guangzhou, China

**Keywords:** diabetes, hypertension, anthropometric indices, central obesity, waist to height ratio (WHtR), cohort study

## Abstract

**Background:**

Patients with comorbidity of hypertension and diabetes are associated with higher morbidity and mortality of cardiovascular disease than those with hypertension or diabetes alone. The present study aimed to identify anthropometric risk factors for diabetes among hypertensive patients who were included in a retrospective cohort study.

**Methods:**

Hypertensive adults without diabetes were recruited in China. Demographic, clinical, biochemical, and anthropometric indices were collected at baseline and during the follow-up. Anthropometric measures included BMI, waist circumference, waist-to-height ratio (WHtR), and waist-to-hip ratio, and several novel indices. To estimate the effect of baseline and dynamic changes of each anthropometric index on risk of new-onset diabetes (defined as self-reported physician-diagnosed diabetes and/or use of hypoglycemic medication, or new-onset FPG≥7.0 mmol/L during follow-up), Cox regression models were used.

**Results:**

A total of 3852 hypertensive patients were studied, of whom 1167 developed diabetes during follow-up. Multivariate Cox regression analyses showed that there was a graded increased risk of incident diabetes with successively increasing anthropometric indices mentioned above (all *P*<0.05). Regardless of the baseline general obesity status, elevated WHtR was both related to higher risk of diabetes; the HRs (95%CI) of baseline BMI<24 kg/m^2^ & WHtR≥0.5 group and BMI≥24 kg/m^2^ & WHtR≥0.5 group were 1.34 (1.05, 1.72), 1.85 (1.48, 2.31), respectively. Moreover, the dynamic changes of WHtR could sensitively reflect diabetes risk. Diabetes risk significantly increased when patients with baseline WHtR<0.5 progressed to WHtR≥0.5 during the follow-up (HR=1.63; 95%CI, 1.11, 2.40). There was also a decreasing trend towards the risk of incident diabetes when baseline abnormal WHtR reversed to normal at follow-up (HR=1.93; 95%CI, 1.36, 2.72) compared with those whose WHtR remained abnormal at follow-up (HR=2.04; 95%CI, 1.54, 2.71).

**Conclusions:**

Central obesity is an independent and modifiable risk factor for the development of diabetes among hypertensive patients. Measuring indices of central obesity in addition to BMI in clinics could provide incremental benefits in the discrimination of diabetes among Chinese hypertensive patients. Dynamic changes of WHtR could sensitively reflect changes in the risk of diabetes. Therefore, long-term monitoring of hypertensive patients using non-invasive anthropometric measures and timely lifestyle intervention could effectively reduce the development of diabetes.

## Introduction

Both hypertension and diabetes represent recognized overall global public health burden (1). Globally, the prevalence of hypertension and diabetes presents a persistently increasing trend; Approximately 1.13 billion and 0.42 billion have hypertension and diabetes, respectively. These two diseases frequently coexist and are closely related, both existing as major risk factors for cardiovascular and cerebrovascular diseases (2). Evidence has revealed that hypertensive patients with diabetes had a two-fold increased risk for developing cardiovascular diseases (CVD) compared with those without diabetes (3). As a result, early recognition of hypertensive patients with a high risk of diabetes is crucial to preventing further progress to cardiovascular and cerebrovascular diseases and improving prognosis.

Obesity is a well-recognized major modifiable risk factor for both diabetes and hypertension. Monitoring the changes in obesity has crucial medical implications for preventing the development of diabetes. Anthropometry is an extensively used, non-invasive, and cost-saving public health tool. Thus, it is of important clinical and public health significance to dig out more effective anthropometric indices related to the onset risk of diabetes among hypertensive patients. BMI is still the most widely used index of obesity for current. Yet, its reliability for determining obesity has been questioned (4–6) since it could not be used to differentiate body composition (fat mass and fat-free mass). Moreover, central obesity has recently received increasing attention because it is more closely correlated with metabolic complications, such as insulin resistance, diabetes, and CVD, than general obesity (7). Importantly, it is associated with an increased risk of diabetes among adults within a healthy BMI range (less than 25kg/m^2^) (8).

Central obesity includes several established and novel parameters. Waist circumferences (WC) and waist-to-hip ratios (WHR) have been frequently used indices in clinical settings. Since WC considers abdominal obesity but ignores the height, another index, waist-to-height ratios (WHtR), has also been used as the alternative anthropometric index for predicting diabetes (9). Nevertheless, comparisons between BMI, WC, and WHtR do not seem to provide sufficient information for anyone of them to have an absolute advantage in predicting diabetes with great sensitivity or specificity (10, 11). In addition, several novel anthropometric indices have been proposed and used: abdominal volume index (AVI) (12), body adiposity index (BAI) (13), body roundness index (BRI) (14), conicity index (CI) (15), and weight-adjusted-waist-index (WWI) (16). Although the mentioned anthropometric indices have been used in various studies, their usefulness has not been systematically evaluated. Furthermore, previous studies mostly are cross-sectional designed and focus on the diabetes risk among general populations (17, 18), lacking concern for the hypertensive patients, especially those with normal-weight central obesity.

Therefore, the present study aimed to compare associations between baseline and changing trends of anthropometric indices with the development of diabetes among hypertensive patients in China. In addition, we examined associations of different combinations of BMI and established measures of central obesity (WC, WHR, and WHtR) with occurrences of diabetes and assessed the magnitude of risk for onset of diabetes from normal BMI with central obesity.

## Methods

### Study Design and Study Population

The retrospective cohort study was conducted in Liaobu Town, Dongguan City, Guangdong Province, China, from 2011 to 2013. The Liaobu Town is a suburb of Dongguan, a well-developed city with a population of 0.42 million, next to the megacity Guangzhou. The subjects were recruited *via* advertisements from the general population in Liaobu community health center hospital using cluster sampling based on the following inclusion criteria: over 18 years old, with hypertension, no history of cancer, willingness to do at least 1-year follow-up. Exclusion criteria were: with self-reported diabetes, unable to respond to interviews, and without valid anthropometric indices or serum biochemical examination at baseline or at least one follow-up. Subsequently, potentially eligible subjects were further assessed based on clinical interviews, health screening questionnaires, physical examination, and fasting-blood analyses, and appropriate subjects were then included for our investigation.

Our study was approved by the Medical Research Ethics Committee of the Guangdong Provincial People’s Hospital (Guangzhou, China). All participants provided written informed consents before their voluntary participation.

### Health Screening Measurements

A structured health screening questionnaire was administered by healthcare staff to each qualified subject and to acquire information on demographics (age, sex, and ethnicities), lifestyle (smoking, alcohol drinking), past medical history (hypertension, diabetes, and dyslipidemia), and current medication uses, if any, for these conditions, and family history of diabetes.

### Health Screening Measurements

All health screening measurements were performed by trained healthcare staff using standard anthropometric techniques. Participants were asked to wear thin clothing with no footwear when taking anthropometric measurements. Body weight, height, WC, and hip circumferences (HC) were each measured twice with the mean recorded. Body weight and height were measured by a standard digital weighing scale and stadiometer, respectively. WC and HC were each measured with the subject standing and during slight expiration using a calibrated tape measure. Waist circumference was measured at the midpoint between the iliac crest and last rib, and hip circumference was measured at the widest part of the hip at the level of the greater trochanter.

Based on the above information, other anthropometric measurements were also collected. BMIs were calculated as weight (kg)/height^2^ (m). WHRs were calculated as WC (m)/HC (m), and WHtRs were calculated as WC (m)/height (m). AVIs were calculated as (2*WC^2^ (cm) + 0.7*(WC (cm)–HC (cm))^2^)/1000 (12). BAIs were calculated as (HC (cm)/height^1.5^ (m))–18 (13). BRIs were calculated as 364.2−365.5*sqrt (1-(WC (m)/(2π))^2^/(0.5*height (m))^2^) (14). CIs were calculated as WC (m)/(0.109*sqrt (weight (kg)/height (m)) (15). WWIs were calculated as WC (cm)/sqrt (weight (kg)) (16) (**Table S1**).

Blood pressure was measured using mercury sphygmomanometers, and participants were required to sit quietly for 5 minutes before the measurement. Hypertension was defined as having systolic blood pressure (SBP)≥140 mmHg, or diastolic blood pressure (DBP)≥ 90mmHg, or with a self-reported history of hypertension, or use of antihypertensive medications.

The health screening measurements as above-mentioned were conducted at baseline and each annual follow-up.

### Laboratory Examinations

Blood samples were collected in the morning after overnight fasting for at least 8 hours. Serum levels of fasting plasma glucose (FPG), triglycerides (TG), total cholesterol (TC), high-density lipoprotein (HDL), low-density lipoprotein (LDL), uric acid (UA), creatinine (Cr), and urinary albumin excretion rate (UAER) were measured *via* a biochemical autonomic analyzer (OLYMPUS, Tokyo, Japan) in the central laboratory, Liaobu community health center hospital. The estimated glomerular filtration rates (eGFR) were calculated using the CKD-EPI creatinine equation (19).

The laboratory measurements as above-mentioned were conducted at baseline and each annual follow-up.

### Obesity Definition

Overweight was defined as having BMI≥24 kg/m^2^ and <28 kg/m2 and obesity as ≥28 kg/m^2^ according to the Working Group on Obesity in China (WGOC) (20). Central obesity was defined as WC≥90 cm for males and WC≥80 cm for females according to the International Diabetes Federation (21), or WHR≥0.90 for males and WHR≥0.85 for females according to WHO guidelines (22). Elevated WHtR was defined as ≥0.5 (9).

Lacking unifying classification standards, cut-off points for novel anthropometric indices (AVI, BAI, BRI, CI, and WWI) were selected at the level of 75% according to the distribution characteristics of BMI in the studied populations (**Table S2**).

### Clinical Outcome

The outcome of the present study was new-onset diabetes, defined as self-reported physician-diagnosed diabetes and/or use of hypoglycemic medication during follow-up, or new-onset FPG≥7.0mmol/L examined at the follow-up examination. All participants were followed until the date of incident diabetes or otherwise until the last follow-up date.

### Statistical Analyses

As estimated in PASS software version 15.0, 390 events would be needed in a Cox regression of the log hazard ratio (HR) to provide 90% power at a 0.05 significance level to detect a regression coefficient equal to 0.20 under an overall event rate of 0.30. For continuous variables, data in line with normal distribution were presented as mean ± standard deviation (SD), while data in line with non-normal distribution were presented as median (1st quartile, 3rd quartile). Categorical data were presented as frequencies (percentages). Differences among the groups were evaluated by the student’s T-test for normally distributed continuous data, by the Kruskal-Wallis rank-sum test for non-normally distributed continuous data, and by the chi-square tests for categorical variables. Univariate Cox regression models were performed to evaluate associations of demographic, biochemical, and clinical characteristics, and anthropometric indices with diabetes. Independent effects of baseline and dynamic changes of each anthropometric index and different combinations among BMI, WC, WHR, and WHtR on the risk of diabetes were estimated using multivariate Cox regression models. Two models with different sets of covariates were fitted. Stratified and interaction analyses were also conducted to evaluate the potential interactions between WHtR and demographic, biochemical and clinical characteristics, and other anthropometric indices. *P*<0.05 (two-tailed) was considered statistically significant. All statistical analyses were conducted using the statistical software packages R 4.0.3 (http://www.R-project.org, The R Foundation).

## Results

### Characteristics of the Participants

**Figure 1:**
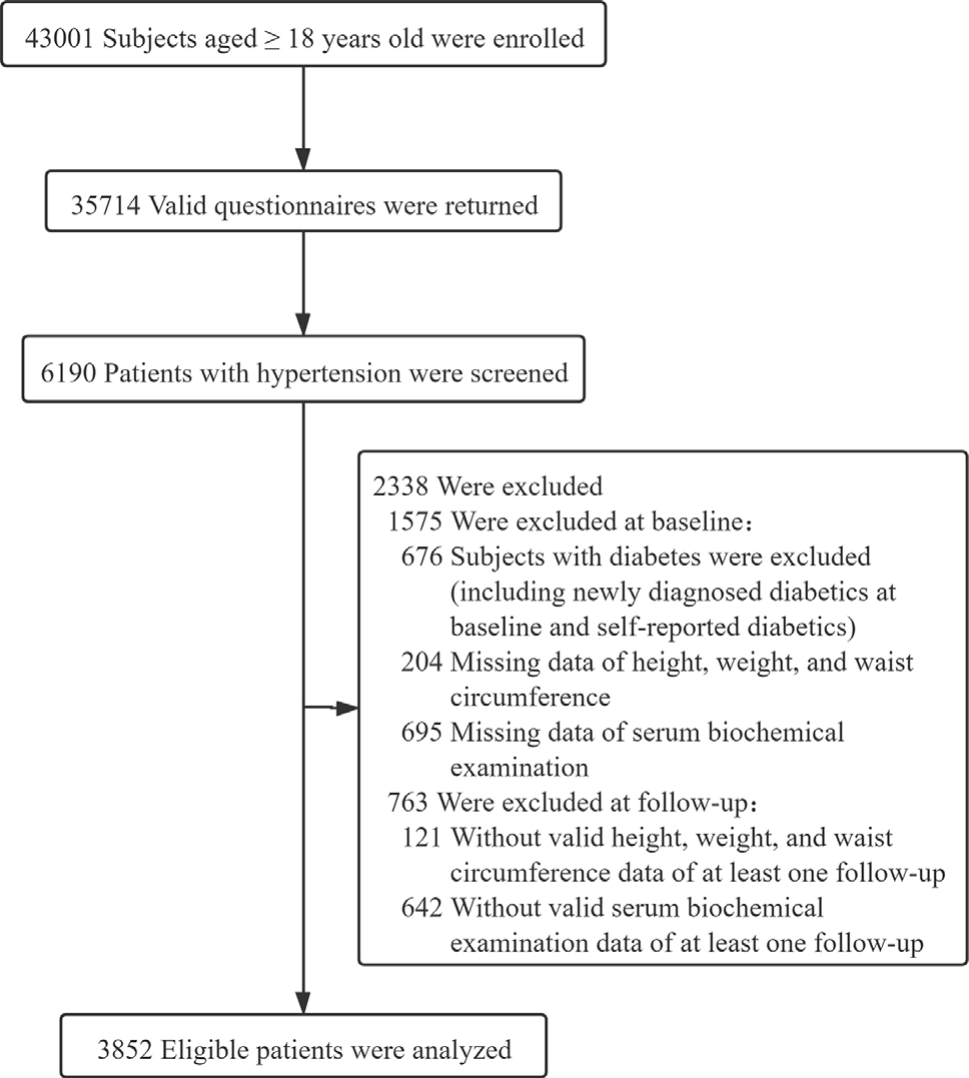
Flow chart of the study population.

From our initial recruitment effort, 43001 subjects aged at least 18 years old underwent the clinical assessment based on clinical interviews, health screening questionnaires, physical examination, and a fasting blood sampling. Among these subjects, 35714 valid questionnaires were returned (83.05% response rate). Based on clinical assessments, 6190 patients with hypertension were included. Finally, after excluding participants with self-reported diabetics or newly diagnosed diabetics at baseline and those who were lost to follow-ups, a total of 3852 participants were included in this analysis (**Figure 1**).

Baseline characteristics of the participants are outlined in **Table 1**. An overall 3852 subjects were studied, out of which 57.7% were females and 42.3% were males. During the median follow-up of 2 years, 1167 participants developed diabetes, 734 were females and 433 were males. Their baseline characteristics as stratified by sex are presented in **Table S3**. The anthropometric indices weight, BMI, WC, WHtR, WHR, AVI, BAI, BRI, CI, and WWI levels were statistically higher in subjects who developed diabetes (*P*<0.001). In addition, compared with subjects who didn’t develop diabetes, those who developed diabetes during follow-up were older, had a higher proportion of females, had higher values of FPG, TG, TC, and SBP, had a higher prevalence of family history of diabetes, while with lower eGFR values and smoking rates (*P*<0.05).

**Table 1:** Baseline characteristics of subjects who did and didn't develop new-onset diabetes during follow-up.

	Total	Diabetes (n = 1167)	Non-diabetes (n = 2685)	*P*-value
Age (years)	61.8 ± 13.6	62.8 ± 12.3	61.3 ± 14.1	0.002^**^
Sex (male (n (%))	1632 (42.37%)	433 (37.10%)	1199 (44.66%)	<0.001^***^
FPG (mmol/L)	5.44 ± 1.68	6.53 ± 2.61	4.97 ± 0.60	<0.001^***^
TG (mmol/L)	2.12 ± 1.71	1.90 (1.32-2.81)	1.61 (1.14-2.27)	<0.001^***^
TC (mmol/L)	5.06 ± 1.26	5.13 ± 1.19	5.03 ± 1.29	0.025^*^
HDL (mmol/L)	1.32 ± 0.36	1.32 ± 0.40	1.32 ± 0.33	0.744
LDL (mmol/L)	2.88 ± 0.80	2.88 ± 0.83	2.88 ± 0.79	0.924
UA (μmol/L)	381.71 ± 104.11	382.70 ± 103.89	381.28 ± 104.22	0.702
Scr (μmol/L)	73.00 (62.00-88.00)	72.00 (61.00-88.00)	73.00 (62.00-88.00)	0.587
eGFR (mL/(min·1.73 m²))	83.62 ± 23.77	81.84 ± 22.43	84.39 ± 24.29	0.002^**^
Weight (kg)	62.63 ± 12.46	64.36 ± 12.04	61.88 ± 12.57	<0.001^***^
BMI (kg/m²)	25.37 ± 3.94	26.19 ± 3.92	25.02 ± 3.90	<0.001^***^
WC (cm)	87.93 ± 9.83	90.22 ± 9.71	86.93 ± 9.71	<0.001^***^
WHtR	0.56 ± 0.07	0.58 ± 0.07	0.56 ± 0.06	<0.001^***^
WHR	0.92 ± 0.06	0.93 ± 0.06	0.92 ± 0.06	<0.001^***^
AVI	15.72 ± 3.48	16.53 ± 3.55	15.37 ± 3.40	<0.001^***^
BAI	30.91 ± 5.34	31.76 ± 5.46	30.54 ± 5.25	<0.001^***^
BRI	4.68 ± 1.43	5.02 ± 1.48	4.54 ± 1.39	<0.001^***^
CI	1.28 ± 0.09	1.30 ± 0.09	1.28 ± 0.09	<0.001^***^
WWI	11.19 ± 0.94	11.31 ± 0.90	11.14 ± 0.95	<0.001^***^
SBP	160.40 ± 22.64	161.62 ± 23.27	159.87 ± 22.35	0.027^*^
DBP	94.54 ± 11.56	94.10 ± 11.72	94.74 ± 11.48	0.119
Smoking (n (%))	743 (19.29%)	190 (16.28%)	553 (20.60%)	0.002^**^
Drinking (n (%))	199 (5.17%)	49 (4.20%)	150 (5.59%)	0.074
Family history of diabetes (n (%))	91 (2.39%)	55 (4.74%)	36 (1.36%)	<0.001^***^

Continuous data are shown as the mean ± SD or median (Q1-Q3), and categorical data as n (%).

FPG, fasting plasma glucose; TG, triglycerides; TC, total cholesterol; HDL, high-density lipoprotein; LDL, low-density lipoprotein; UA, urid acid; Scr, serum creatinine; eGFR, estimated glomerular filtration rate; BMI, body mass index; WC, waist circumference; WHtR, waist-to-height ratio; WHR, waist-to-hip ratio; AVI, abdominal volume index; BAI, body adiposity index; BRI, body roundness index; CI, conicity index; WWI, weight-adjusted-waist index; SBP, systolic blood pressure; DBP, diastolic blood pressure.

^*^P-value < 0.05; ^**^P-value < 0.01; ^***^P-value < 0.001.

### Correlations Between Baseline Anthropometric Measures and the Development of Diabetes in Follow-Ups

Correlations between the baseline clinical variables and new-onset diabetes are displayed in **Table S4**. The univariate Cox regression analyses revealed that the development of diabetes was positively correlated with age, sex, TG, TC, and family history of diabetes, and negatively correlated with smoking status and eGFR (*P*<0.05).

After fully adjustment for sex, age, smoking status, drinking status, and family history of diabetes at baseline, and differences of FPG, TG, TC, HDL, LDL, SBP, DBP between the baseline and follow-up, the elevation of all indices analyzed in this study were each independently associated with an increase in risk of incident diabetes (**Tables 2** and **S5**): elevated weight (HR=1.41; 95%CI: 1.31, 1.51; *P*<0.001), BMI (HR=1.32; 95%CI: 1.25, 1.40; *P*<0.001), WC (HR=1.32; 95%CI: 1.25, 1.40; *P*<0.001), WHtR (HR=1.27; 95%CI: 1.20, 1.36; *P*<0.001), WHR (HR=1.21; 95%CI: 1.15, 1.28; *P*<0.001), AVI (HR=1.31; 95%CI: 1.23, 1.38; *P <*0.001), BAI (HR=1.15; 95%CI: 1.07, 1.23; *P*<0.001), BRI (HR=1.26; 95%CI: 1.19, 1.33; *P <*0.001), CI (HR=1.14; 95%CI: 1.07, 1.22; *P*<0.001), WWI (HR=1.11; 95%CI: 1.04, 1.19; *P*=0.003).

**Table 2:** Multivariate cox regression models evaluating the associations of baseline established anthropometric indices with the development of diabetes.

	Unadjusted model		Model 1		Model 2	
	HR (95%CI)	*P*-value	HR (95%CI)	*P*-value	HR (95%CI)	*P*-value
**Weight (kg)**						
As continuous variables (per SD increment)	1.18 (1.12, 1.25)	<0.001^***^	1.44 (1.35, 1.54)	<0.001^***^	1.41 (1.31, 1.51)	<0.001^***^
<75 in males or <65 in females	1.0		1.0		1.0	
≥75 in males or ≥65 in females	1.40 (1.24, 1.59)	<0.001^***^	1.56 (1.37, 1.77)	<0.001^***^	1.52 (1.33, 1.74)	<0.001^***^
**BMI (kg/m²)**						
As continuous variables (per SD increment)	1.27 (1.21, 1.34)	<0.001^***^	1.33 (1.26, 1.40)	<0.001^***^	1.32 (1.25, 1.40)	<0.001^***^
<24.0	1.0		1.0		1.0	
24.0-28.0	1.40 (1.22, 1.61)	<0.001^***^	1.46 (1.27, 1.69)	<0.001^***^	1.37 (1.19, 1.59)	<0.001^***^
≥28.0	1.80 (1.54, 2.09)	<0.001^***^	1.92 (1.64, 2.24)	<0.001^***^	1.79 (1.53, 2.11)	<0.001^***^
**WC (cm)**						
As continuous variables (per SD increment)	1.34 (1.26, 1.41)	<0.001^***^	1.36 (1.28, 1.44)	<0.001^***^	1.32 (1.25, 1.40)	<0.001^***^
<90 in males or <80 in females	1.0		1.0		1.0	
≥90 in males or ≥80 in females	1.64 (1.44, 1.88)	<0.001^***^	1.62 (1.41, 1.87)	<0.001^***^	1.51 (1.30, 1.74)	<0.001^***^
**WHtR**						
As continuous variables (per SD increment)	1.33 (1.26, 1.41)	<0.001^***^	1.31 (1.24, 1.39)	<0.001^***^	1.27 (1.20, 1.36)	<0.001^***^
<0.5	1.0		1.0		1.0	
≥0.5	1.80 (1.48, 2.19)	<0.001^***^	1.72 (1.41, 2.10)	<0.001^***^	1.61 (1.32, 1.98)	<0.001^***^
**WHR**						
As continuous variables (per SD increment)	1.22 (1.16, 1.28)	<0.001^***^	1.23 (1.16, 1.29)	<0.001^***^	1.21 (1.15, 1.28)	<0.001^***^
<0.90 in males or <0.85 in females	1.0		1.0		1.0	
≥0.90 in males or ≥0.85 in females	1.61 (1.37, 1.90)	<0.001^***^	1.54 (1.30, 1.82)	<0.001^***^	1.47 (1.24, 1.75)	<0.001^***^

BMI, body mass index; WC, waist circumference; WHtR, waist-to-height ratio; WHR, waist-to-hip ratio.

Model 1: adjusted by sex, age, smoking status, drinking status, and family history of diabetes at baseline.

Model 2: adjusted by model 1 plus differences of FPG, TG, TC, HDL, LDL, SBP, DBP between the baseline and follow-up.

^***^P -value < 0.001.

**Figure 2:**
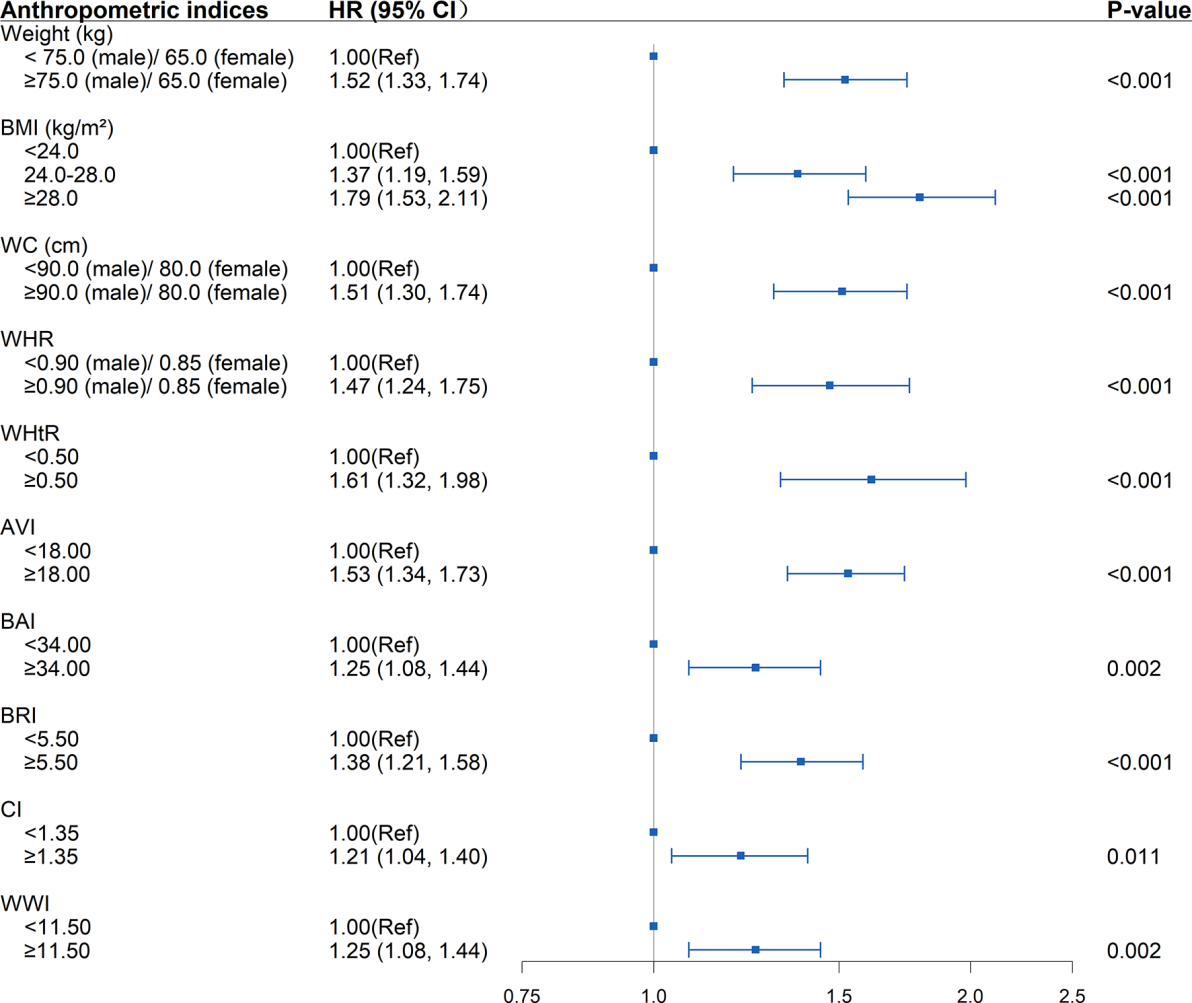
Association between separate anthropometric indices with the development of diabetes (weight, body mass index [BMI], waist circumference [WC], waist-to-hip ratio [WHR], waist-to-height ratio [WHtR], abdominal volume index [AVI], body adiposity index [BAI], body roundness index [BRI], conicity index [CI], weight-adjusted-waist index [WWI]). The correlation was assessed by multivariate cox regression analysis, adjusted by sex, age, smoking status, drinking status, and family history of diabetes at baseline and differences of FPG, TG, TC, HDL, LDL, SBP, DBP between the baseline and follow-up. Hazard ratios (HRs) of the anthropometric indices were represented as the squares and 95% confidence intervals (CIs) by the lines through the squares.

Adopting internationally recognized diagnostic for central obesity, there was a graded increased risk of incident diabetes with successively increasing WC (HR=1.51; 95%CI: 1.30, 1.74; *P*<0.001), WHtR (HR=1.61; 95%CI: 1.32, 1.98; *P*<0.001) and WHR (HR=1.47; 95%CI: 1.24, 1.75; *P*<0.001). Moreover, selecting the level of 75% for the novel anthropometric indices as the cut-off point, the new-onset diabetes risk increased when any of the following conditions was met: AVI≥18 (HR=1.53; 95%CI: 1.34, 1.73 *P*<0.001), BAI≥34 (HR=1.25; 95%CI: 1.08, 1.44; *P*=0.002), BRI≥5.5 (HR=1.38; 95%CI: 1.21, 1.58; *P*<0.001), CI≥1.35 (HR=1.21; 95%CI: 1.04, 1.40; *P*<0.001), WWI≥11.5 (HR=1.25; 95%CI: 1.08, 1.44; *P*=0.002) (**Figure 2**).

**Figure 3:**
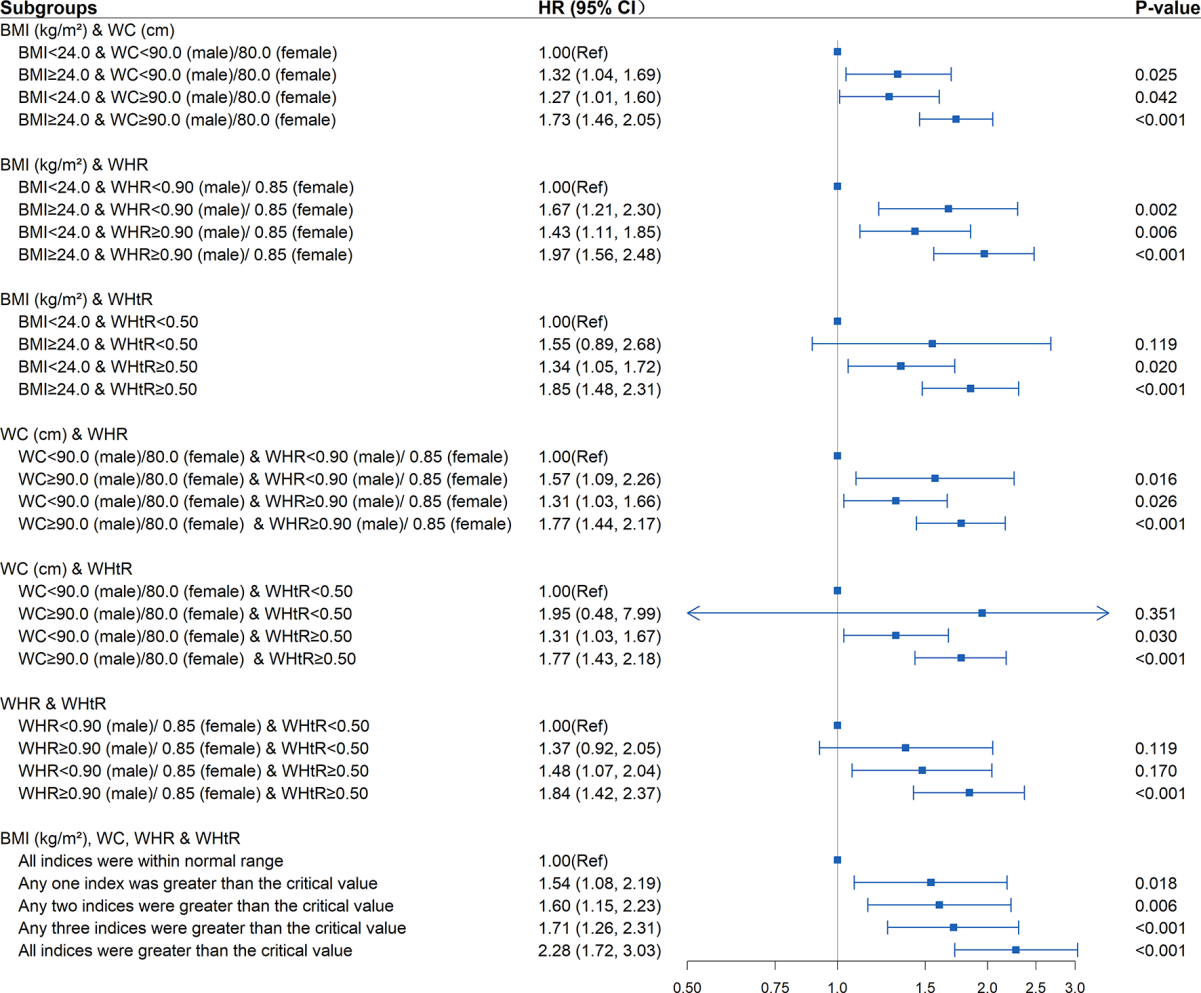
Association between different combinations of body mass index (BMI) and established anthropometric indices of central obesity (waist circumference [WC], waist-to-hip ratio [WHR], waist-to-height ratio [WHtR]) with the development of diabetes. The correlation was assessed by multivariate cox regression analysis, adjusted by sex, age, smoking status, drinking status, and family history of diabetes at baseline and differences of FPG, TG, TC, HDL, LDL, SBP, DBP between the baseline and follow-up. Hazard ratios (HRs) of the combined anthropometric indices were represented as the squares and 95% confidence intervals (CIs) by the lines through the squares.

New-onset diabetes risk increased significantly with the baseline WHtR levels above 0.5 whether the BMI, WC, and WHR were within the normal range (BMI<24kg/m^2^, WC<90cm in males or <80cm in females, WHR<0.90 in males or <0.85 in females) or not at baseline. Elevated WHtR (WHtR≥0.5) at baseline was significantly associated with increased diabetes risk with baseline BMI<24kg/m^2^ (HR=1.37; 95%CI: 1.08, 1.75; P =0.011), WC<90cm in males or <80cm in females (HR=1.34; 95%CI: 1.06, 1.71; P =0.015), WHR<0.90 in males or <0.85 in females (HR=1.62; 95%CI: 1.18, 2.22; *P*=0.003). The risk increased further when BMI>24kg/m^2^ (HR=1.85; 95%CI: 1.48, 2.31; *P*<0.001), WC≥90cm in males or ≥80cm in females (HR=1.77; 95%CI: 1.43, 2.18; *P*<0.001), and WHR≥0.90 in males or ≥0.85 in females (HR=1.84; 95%CI: 1.42, 2.37; *P*<0.001) at baseline. The highest risk for incident diabetes (HR=2.28; 95%CI: 1.72, 3.03; *P*<0.001) was observed when BMI, WC, WHR, WHtR were all greater than the critical value (**Table 3** and **Figure 3**). Interaction and stratified analyses revealed that there were no significant interactions between WHtR and other clinical variables (**Table S7**).

**Table 3:** Multivariate cox regression models evaluating the associations of different combinations of BMI and established anthropometric indices of central obesity (WC, WHR and WHtR) with the development of diabetes.

	Unadjusted model		Model 1		Model 2	
	HR (95%CI)	*P*-value	HR (95%CI)	*P*-value	HR (95%CI)	*P*-value
**BMI (kg/m^2^) & WC (cm)**						
BMI<24 & WC<90 (male)/80 (female)	1.0		1.0		1.0	
BMI≥24 & WC<90 (male)/80 (female)	1.26 (1.00, 1.60)	0.053	1.34 (1.05, 1.71)	0.017^*^	1.32 (1.04, 1.69)	0.025^*^
BMI<24 & WC≥90 (male)/80 (female)	1.42 (1.14, 1.76)	0.002^**^	1.31 (1.05, 1.64)	0.019^*^	1.27 (1.01, 1.60)	0.042^*^
BMI≥24 & WC≥90 (male)/80 (female)	1.87 (1.59, 2.19)	<0.001^***^	1.88 (1.60, 2.22)	<0.001^***^	1.73 (1.46, 2.05)	<0.001^***^
**BMI (kg/m^2^) & WHtR**						
BMI<24 & WHtR<0.5	1.0		1.0		1.0	
BMI≥24 & WHtR<0.5	1.39 (0.82, 2.37)	0.219	1.61 (0.94, 2.74)	0.081	1.55 (0.89, 2.68)	0.119
BMI<24 & WHtR≥0.5	1.50 (1.18, 1.90)	<0.001^***^	1.37 (1.08, 1.75)	0.011^*^	1.34 (1.05, 1.72)	0.020^*^
BMI≥24 & WHtR≥0.5	2.03 (1.64, 2.52)	<0.001^***^	2.00 (1.61, 2.48)	<0.001^***^	1.85 (1.48, 2.31)	<0.001^***^
**BMI (kg/m^2^) & WHR**						
BMI<28 & WHR<0.90 (male)/0.85 (female)	1.0		1.0		1.0	
BMI≥28 & WHR<0.90 (male)/0.85 (female)	1.62 (1.19, 2.19)	0.002^**^	1.72 (1.26, 2.34)	<0.001^***^	1.67 (1.21, 2.30)	0.002^**^
BMI<28 & WHR≥0.90 (male)/0.85 (female)	1.56 (1.22, 2.00)	<0.001^***^	1.43 (1.11, 1.84)	0.005^**^	1.43 (1.11, 1.85)	0.006^**^
BMI≥28 & WHR≥0.90 (male)/0.85 (female)	2.16 (1.73, 2.70)	<0.001^***^	2.10 (1.68, 2.64)	<0.001^***^	1.97 (1.56, 2.48)	<0.001^***^
**WC (cm) & WHtR**						
WC<90 (male)/80 (female) & WHtR<0.5	1.0		1.0		1.0	
WC≥90 (male)/80 (female) & WHtR<0.5	2.90 (0.92, 9.13)	0.069	2.40 (0.76, 7.62)	0.137	1.95 (0.48, 7.99)	0.351
WC<90 (male)/80 (female) & WHtR≥0.5	1.38 (1.09, 1.74)	0.008^**^	1.34 (1.06, 1.71)	0.015^*^	1.31 (1.03, 1.67)	0.030^*^
WC≥90 (male)/80 (female) & WHtR≥0.5	1.99 (1.63, 2.43)	<0.001^***^	1.93 (1.57, 2.37)	<0.001^***^	1.77 (1.43, 2.18)	<0.001^***^
**WC (cm) & WHR**						
WC<90 (male)/80 (female) & WHR<0.90 (male)/0.85 (female)	1.0		1.0		1.0	
WC≥90 (male)/80 (female) & WHR<0.90 (male)/0.85 (female)	1.62 (1.16, 2.28)	0.005^**^	1.66 (1.17, 2.37)	0.005^**^	1.57 (1.09, 2.26)	0.016^*^
WC<90 (male)/80 (female) & WHR≥0.90 (male)/0.85 (female)	1.34 (1.06, 1.69)	0.013^*^	1.31 (1.04, 1.65)	0.023^*^	1.31 (1.03, 1.66)	0.026^*^
WC≥90 (male)/80 (female) & WHR≥0.90 (male)/0.85 (female)	1.96 (1.62, 2.37)	<0.001^***^	1.90 (1.56, 2.32)	<0.001^***^	1.77 (1.44, 2.17)	<0.001^***^
**WHR & WHtR**						
WHR<0.90 (male)/0.85 (female) & WHtR<0.5	1.0		1.0		1.0	
WHR≥0.90 (male)/0.85 (female) & WHtR<0.5	1.53 (1.05, 2.24)	0.028^*^	1.47 (1.00, 2.16)	0.049^*^	1.37 (0.92, 2.05)	0.119
WHR<0.90 (male)/0.85 (female) & WHtR≥0.5	1.65 (1.21, 2.25)	0.002^**^	1.62 (1.18, 2.22)	0.003^***^	1.48 (1.07, 2.04)	0.017^*^
WHR≥0.90 (male)/0.85 (female) & WHtR≥0.5	2.15 (1.68, 2.76)	<0.001^***^	2.02 (1.57, 2.60)	<0.001	1.84 (1.42, 2.37)	<0.001^***^
**BMI, WC, WHtR & WHR**						
All indicators were normal	1.0		1.0		1.0	
Any one indicator was abnormal	1.61 (1.14, 2.28)	0.007^**^	1.60 (1.13, 2.27)	0.008^**^	1.54 (1.08, 2.19)	0.018^*^
Any two indicators were abnormal	1.71 (1.23, 2.36)	0.001^**^	1.65 (1.19, 2.29)	0.003^**^	1.60 (1.15, 2.23)	0.006^**^
Any three indicators were abnormal	1.91 (1.42, 2.57)	<0.001^***^	1.81 (1.35, 2.45)	<0.001^***^	1.71 (1.26, 2.31)	<0.001^***^
All indicators were abnormal	2.61 (1.98, 3.45)	<0.001^***^	2.54 (1.92, 3.36)	<0.001^***^	2.28 (1.72, 3.03)	<0.001^***^

BMI, body mass index; WC, waist circumference; WHtR, waist-to-height ratio; WHR, waist-to-hip ratio.

Model 1: adjusted by sex, age, smoking status, drinking status, and family history of diabetes at baseline.

Model 2: adjusted by model 1 plus differences of FPG, TG, TC, HDL, LDL, SBP, DBP between the baseline and follow-up.

^*^P-value < 0.05; ^**^P -value < 0.01; ^***^P -value < 0.001.

### Correlations Between Dynamic Changes of Anthropometric Measures and the Development of Diabetes During Follow-Ups

**Figure 4:**
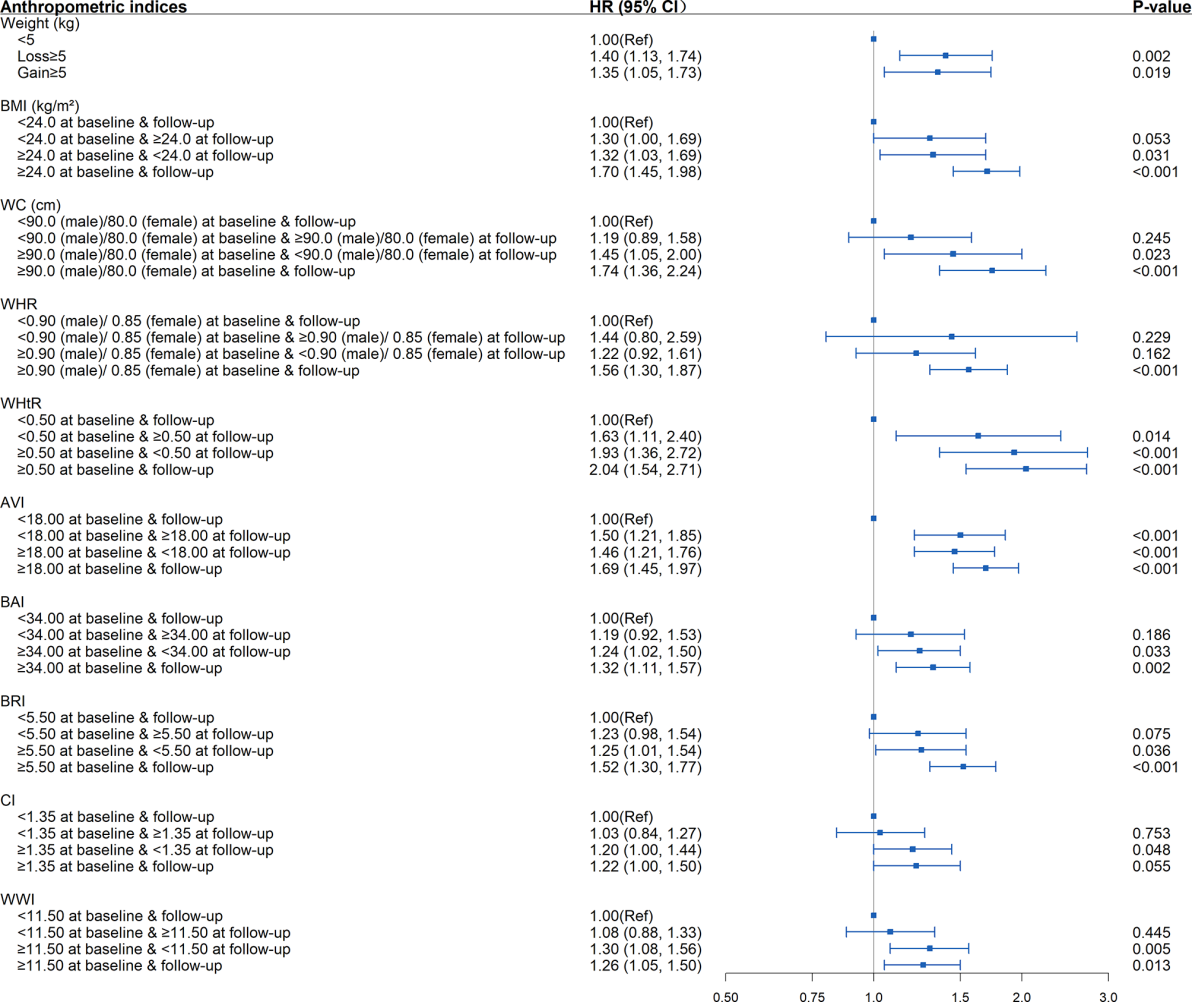
Association between dynamic changes of separate anthropometric indices with the development of diabetes (weight, body mass index [BMI], waist circumference [WC], waist-to-hip ratio [WHR], waist-to-height ratio [WHtR], abdominal volume index [AVI], body adiposity index [BAI], body roundness index [BRI], conicity index [CI], weight-adjusted-waist index [WWI]). The correlation was assessed by multivariate cox regression analysis, adjusted by sex, age, smoking status, drinking status, and family history of diabetes at baseline and differences of FPG, TG, TC, HDL, LDL, SBP, DBP between the baseline and follow-up. Hazard ratios (HRs) of the anthropometric indices were represented as the squares and 95% confidence intervals (CIs) by the lines through the squares.

As shown in **Tables 4**, **S6**, and **Figure 4**, in the fully adjusted model, compared with the subjects whose WHtR was less than 0.5 at baseline and follow-up, elevated WHtR (WHtR≥0.5) at baseline or follow-up was associated with a higher risk of developing diabetes (*P*<0.05). When WHtR≥0.5 was detected at baseline, the risk of diabetes tended to be higher in subjects with WHtR≥0.5 at follow-up (HR=2.04; 95%CI: 1.54, 2.71; *P*<0.001) than those whose WHtR returned to less than 0.5 (HR=1.93; 95%CI: 1.36, 2.72; *P*<0.001). The highest risk of diabetes onset was observed when WHtR≥0.5 both at baseline and follow-up. Among the subjects with BMI within the normal range at the baseline, compared with the subjects who remained BMI<24kg/m^2^ during the follow-up, subjects whose BMI became overweight or obese at follow-up were not at a significant increase in risk for diabetes. The same was observed in WC and WHR.

**Table 4:** Multivariate cox regression models evaluating the associations of dynamic changes of established anthropometric indices with the development of diabetes.

	Unadjusted model		Model 1		Model 2	
	HR (95%CI)	*P*-value	HR (95%CI)	*P*-value	HR (95%CI)	*P*-value
**Weight change (kg)**						
<5	1.0		1.0		1.0	
Loss ≥5	1.32 (1.12, 1.55)	<0.001^***^	1.33 (1.13, 1.56)	<0.001^***^	1.40 (1.13, 1.74)	0.002^**^
Gain ≥5	1.47 (1.23, 1.76)	<0.001^***^	1.45 (1.21, 1.74)	<0.001^***^	1.35 (1.05, 1.73)	0.019^*^
**Dynamic changes of BMI (kg/m^2^)**						
<24 at baseline & <24 at follow-up	1.0		1.0		1.0	
<24 at baseline & ≥24 at follow-up	1.38 (1.08, 1.76)	0.010^***^	1.36 (1.07, 1.74)	0.014^*^	1.30 (1.00, 1.69)	0.053
≥24 at baseline & <24 at follow-up	1.32 (1.04, 1.68)	0.023^*^	1.35 (1.06, 1.72)	0.014^*^	1.32 (1.03, 1.69)	0.031^*^
≥24 at baseline & ≥24 at follow-up	1.68 (1.45, 1.95)	<0.001^***^	1.76 (1.51, 2.05)	<0.001^***^	1.70 (1.45, 1.98)	<0.001^***^
**Dynamic changes of WC (cm)**						
<90 (male)/80 (female) at baseline & <90 (male)/80 (female) at follow-up	1.0		1.0		1.0	
<90 (male)/80 (female) at baseline & ≥90 (male)/80 (female) at follow-up	1.27 (0.98, 1.65)	0.068	1.35 (1.03, 1.76)	0.029^*^	1.19 (0.89, 1.58)	0.245
≥90 (male)/80 (female) at baseline & <90 (male)/80 (female) at follow-up	1.59 (1.18, 2.14)	0.002^**^	1.60 (1.19, 2.16)	0.002^**^	1.45 (1.05, 2.00)	0.023^*^
≥90 (male)/80 (female) at baseline & ≥90 (male)/80 (female) at follow-up	1.99 (1.58, 2.51)	<0.001^***^	2.00 (1.58, 2.53)	<0.001^***^	1.74 (1.36, 2.24)	<0.001^***^
**Dynamic changes of WHtR**						
<0.5 at baseline & <0.5 at follow-up	1.0		1.0		1.0	
<0.5 at baseline & ≥0.5 at follow-up	1.80 (1.23, 2.64)	0.003^**^	1.71 (1.16, 2.51)	0.006^**^	1.63 (1.11, 2.40)	0.014^*^
≥0.5 at baseline & <0.5 at follow-up	2.02 (1.44, 2.84)	<0.001^***^	1.98 (1.41, 2.78)	<0.001^***^	1.93 (1.36, 2.72)	<0.001^***^
≥0.5 at baseline & ≥0.5 at follow-up	2.33 (1.77, 3.07)	<0.001^***^	2.18 (1.65, 2.88)	<0.001^***^	2.04 (1.54, 2.71)	<0.001^***^
**Dynamic changes of WHR**						
<0.90 (male)/0.85 (female) at baseline & <0.90 (male)/0.85 (female) at follow-up	1.0		1.0		1.0	
<0.90 (male)/0.85 (female) at baseline & ≥0.90 (male)/0.85 (female) at follow-up	1.33 (0.75, 2.34)	0.330	1.30 (0.72, 2.33)	0.390	1.44 (0.80, 2.59)	0.229
≥0.90 (male)/0.85 (female) at baseline & <0.9 (male)/0.85 (female) at follow-up	1.22 (0.93, 1.60)	0.158	1.21 (0.92, 1.60)	0.164	1.22 (0.92, 1.61)	0.162
≥0.90 (male)/0.85 (female) at baseline & ≥0.90 (male)/0.85 (female) at follow-up	1.69 (1.42, 2.01)	<0.001^***^	1.60 (1.34, 1.92)	<0.001^***^	1.56 (1.30, 1.87)	<0.001^***^

BMI, body mass index; WC, waist circumference; WHtR, waist-to-height ratio; WHR, waist-to-hip ratio.

Model 1: adjusted by sex, age, smoking status, drinking status, and family history of diabetes at baseline.

Model 2: adjusted by model 1 plus differences of FPG, TG, TC, HDL, LDL, SBP, DBP between the baseline and follow-up.

^*^P-value < 0.05; ^**^P-value < 0.01; ^***^P-value < 0.001.

Other significant associations between the changing trends in anthropometric indices and the development of diabetes included elevated AVI (AVI≥18) at baseline and/or follow-up, elevated BAI, BRI, CI, WWI at baseline (BAI≥34, BRI≥5.5, CI≥1.35, WWI≥11.5) regardless of returning to less than the critical value during follow-up or not, weight loss ≥5 kg, and weight gain ≥5 kg (*P*<0.05).

## Discussion

Diabetes and hypertension frequently coexist in patients, and both are established risk factors for CVD (2, 23). Individuals with diabetes and hypertension have higher morbidity and mortality of CVD compared with those with either disease alone (3). Therefore, it is crucial to explore and intervene in the risk factors for developing diabetes in the hypertension population. Hypertension could be easily identified by non-invasive BP measurements, yet diabetes often goes undetected until patients present with diabetic complications. Anthropometric measurements have been widely used in clinical screening for CVD and metabolic syndrome (MetS), owing to their simple, low-cost, quick, and non-invasive characteristics. The present study was conducted to compare the strength of associations between different anthropometric indices with the development of diabetes in the hypertension population.

In the present cohort of patients with hypertension, our data indicate that anthropometric measurements analyzed in this study, BMI, WC, WHtR, WHR, AVI, BAI, BRI, CI, and WWI, were each independently associated with increased risk for the development of diabetes. Among these indices, WHtR had the strongest association with the new-onset diabetic risk, with dynamic changes showing stronger associations than BMI, WC, and WHR. Additionally, regardless of whether the BMI, WC, and WHR were within the normal range or not, elevated WHtR at baseline was associated with an increased risk of diabetes. Our results were consistent with a previous robust meta-analysis which shows that measures of abdominal obesity were better indicators for obesity-related cardiometabolic risk than BMI, and WHtR was a better screening tool than WC and BMI for diabetes, hypertension, and CVD (9). Previous studies have considered mechanisms to explain why measures of central obesity are better than BMI in predicting diabetes. Yet, there are few related studies on why should WHtR be superior to WC. We speculate that on the one hand, for the adults whose height is generally stable, the change of WHtR is essentially the change in WC, whereas WHtR partially precludes the influence of age and sex. On the other hand, adverse early life exposures lead to short stature and are also closely associated with predisposition to abdominal obesity and insulin resistance in adults (9), which is biologically plausible.

For identification of central obesity, WC, WHR, and WHtR measurements have often been used. Our data indicated that for the population with hypertension, normal weight but central obesity was also associated with elevated risk for diabetes. Our finding is consistent with a previous report that Asian populations are susceptible to develop diabetes despite having relatively lower BMI than other ethnicities (24). This indicates that abdominal obesity may be a more useful indicator than BMI for diabetes, especially for hypertensive patients. Several potential mechanisms could be used to explain our findings. To begin with, ectopic fat accumulation, whose marker is abdominal fat, has been confirmed to increase the risk of metabolic abnormality and future development of diabetes (25, 26). Additionally, compared with subcutaneous fat, visceral fat with abdominal cavities is related to higher metabolic and inflammatory activities, thus prompting the development of diabetes (27). Further, normal weight with central obesity indicates that such individuals have excessive visceral fat, and their normal BMI usually means they are at higher risk of less muscle compared with the same BMI but no central obesity. And the lack of muscle mass has been confirmed to be associated with adverse metabolic profiles (28). At the same time, it’s interesting to note that the increased risk of diabetes among hypertensive patients with overweight/obesity but with WHtR<0.5 was not statistically significant, further revealing that the development of diabetes is more closely related to the distribution rather than the absolute degree of adiposity per se. Therefore, the indices of central obesity could be measured in addition to BMI to identify the patients with normal-weight central obesity who are also at high risk of diabetes, and thus provide incremental benefit in the pre-screening of hypertensive patients with diabetes.

Our study revealed a trend to reduce the onset risk of diabetes when WC, WHR, and WHtR were reversed towards normal from abnormal levels. In addition, the diabetes risk could also be observed to be increased with increasing WHtR during follow-up. Of note, the changing trends of AVI performed similarly in reflecting the risk of T2DM as WHtR, showing superior sensitivity of both its baseline value and dynamic changes on reflecting the development than other indices, which suggests that AVI could also be effective predictive indicators of diabetes. It’s well documented that the development of diabetes could be delayed or prevented through lifestyle intervention, including dietary modification, weight loss, and exercise training. Therefore, through long-term monitoring of these non-invasive and straightforward measures and applying timely lifestyle intervention, it’s expected to promote the switch from abnormal towards normal levels of these established or novel indices of central obesity, which is essential for preventing or delaying the development of diabetes.

Our study has important implications for public health and clinical practice. First, according to the current established guidelines, individuals with normal weight based on BMI, regardless of central obesity status, were generally regarded as normal in clinical practice. This could lead to a missed opportunity for timely evaluation and intervention for a subgroup that is at high risk but easily neglected (i.e., those with normal-weight central obesity). Second, since all the anthropometric indices of central obesity were calculated based on WC, which could be easily implemented in different levels of hospitals with only a tape used and simple standardized training of the healthcare personnel, WC is recommended to be routinely obtained in daily clinical practice. Third, our findings suggested that the dynamic changes of WC, WHR, and WHtR could sensitively reflect the variation of diabetes onset risk. Thus, the decrease in WC should be the vital focus for public health preventive interventions for diabetes since the height remains nearly unchanged.

The following limitations should be taken into account when interpreting our findings. First, this was a monocentric study. Although the study was conducted in a representative population in China, our findings might not be extrapolated to other populations in China or Asia. Second, some known risk factors for diabetes, such as dietary habits and physical activity status, were not collected, and their impact on our study could not be adjusted. Third, due to the lack of uniform criteria for the novel anthropometric indices in the Chinese population, the 75% value was selected as the cut-off point to explore the association with diabetes risk in the present study. Fourth, the retrospective nature of the study is a limitation since we could not account for those with missing data, and those lost to follow-up (including deaths). Last, the diagnosis of new diabetes is based on suboptimal criteria. Therefore, further studies with larger sample size and multicenter design are needed to confirm our findings. Despite these limitations, our study still has some important strengths, including the cohort study design, which could establish the temporal sequencing of a causal association. Additionally, except for the baseline values of anthropometric indices, we also examined the associations of different combinations of BMI and these indices and changing trends of these indices with diabetes risk, providing deeper insights into the role of central obesity on diabetes. Last, the anthropometric measures were collected directly by the trained healthcare personnel instead of self-reported by the participants.

In conclusion, our study has demonstrated that central obesity is a significant, independent, and modifiable risk factor for diabetes among the population with hypertension. Measuring indices of central obesity, especially WHtR, in addition to BMI in clinics could provide incremental benefits in the discrimination of diabetes in Chinese hypertensive patients. Moreover, we suggest that AVI might be a promising predictor for diabetes screening.

## Data Availability Statement

The raw data supporting the conclusions of this article will be made available by the authors, upon reasonable request.

## Ethics Statement

Our study was approved by the Medical Research Ethics Committee of the Guangdong Provincial People’s Hospital (Guangzhou, China). All participants provided written informed consents before their voluntary participation.

## Author Contributions

All authors have contributed to the creation of this manuscript for important intellectual content. Conceptualization, JK and HG. Methodology, JK, HC, and HG. Validation, YX and QH. Formal Analysis, YL and XL. Investigation, XF. Resources, QZ. Data Curation, SZ. Writing — original draft preparation, YL. Writing – review and editing, JK and HC. Visualization, XL. Supervision, JK. Project administration, JK. Funding acquisition, JK and HC. All authors contributed to the article and approved the submitted version.

## Funding

This work was supported by the National Key R&D Program of China under Grant No. 2018YFC1314100, the Key-Area Research and Development Program of Guangdong Province under Grant No. 2019B020230001, and the Science and Technology Plan of Guangzhou under Grant No. 201707010330.

## Conflict of Interest

The authors declare that the research was conducted in the absence of any commercial or financial relationships that could be construed as a potential conflict of interest.

## Publisher’s Note

All claims expressed in this article are solely those of the authors and do not necessarily represent those of their affiliated organizations, or those of the publisher, the editors and the reviewers. Any product that may be evaluated in this article, or claim that may be made by its manufacturer, is not guaranteed or endorsed by the publisher.
