# Supplementary material for: Association of Anthropometric Indices With the Development of Diabetes Among Hypertensive Patients in China: A Cohort Study
Source: Front Endocrinol (Lausanne). 2021 Oct 5;12:736077. doi: 10.3389/fendo.2021.736077 (PMC8525507; doi:10.3389/fendo.2021.736077)
Supplement: Supplementary file 1 [file Table_1.pdf]

Supplementary Material

1 Supplementary Figures and Tables

Table S1 Formulas of anthropometric indices.

Anthropometric indices	Formula
Body mass index (BMI)	$BMI = Weight_{(kg)} \div Height^2{}_{(m)}$
Waist-to-hip ratio (WHR)	$WHR = WC_{(m)} \div HC_{(m)}$
waist-to-height ratio (WHtR)	$WHtR = WC_{(m)} \div Height_{(m)}$
Abdominal volume index (AVI)	$AVI = \frac{2 \times WC^2{}_{(cm)} + 0.7 \times (WC_{(cm)} - HC_{(cm)})^2}{1000}$
Body adiposity index (BAI)	$BAI = HC_{(cm)} \div Height^{1.5}{}_{(m)} - 18$
Body roundness index (BRI)	$BRI = 364.2 - 365.5 \times \sqrt{1 - \left(\frac{WC_{(m)}}{2\pi}\right)^2 \div (0.5 \times Height_{(m)})^2}$
Conicity index (CI)	$CI = \frac{WC_{(m)}}{0.109 \times \sqrt{\frac{Weight_{(kg)}}{Height_{(m)}}}}$
Weight-adjusted-waist-index (WWI)	$WWI = WC_{(cm)} \div Weight^{\frac{1}{2}}{}_{(kg)}$

Abbreviations: BMI, body mass index; WC, waist circumference; HC, hip circumference; WHtR, waist-to-height ratio; WHR, waist-to-hip ratio; AVI, abdominal volume index; BAI, body adiposity index; BRI, body roundness index; CI, conicity index; WWI, weight-adjusted-waist index.

Table S2 Distribution characteristics of anthropometric indices among participants studied.

	Sex	25%	50%	75%	90%	95%
Weight (kg)	Male	60.00	68.00	75.00	83.00	87.50
	Female	51.00	58.00	65.00	72.00	77.00
	Total	54.00	62.00	70.50	78.00	84.00
BMI (kg/m^2)	Male	22.96	25.21	27.55	29.92	31.24
	Female	22.77	25.15	27.77	30.59	32.41
	Total	22.84	25.15	27.68	30.34	31.96
WC (cm)	Male	82.00	88.00	94.00	100.00	104.00
	Female	81.00	88.00	94.00	100.00	104.00
	Total	82.00	88.00	94.00	100.00	104.00
WHtR	Male	0.50	0.53	0.57	0.61	0.63
	Female	0.53	0.58	0.62	0.66	0.69
	Total	0.52	0.56	0.60	0.65	0.68
WHR	Male	0.89	0.93	0.96	0.99	1.01
	Female	0.88	0.91	0.96	0.99	1.02
	Total	0.88	0.92	0.96	0.99	1.01
AVI	Male	13.57	15.59	17.81	20.00	21.66
	Female	13.21	15.49	17.71	20.06	21.66
	Total	13.48	15.52	17.73	20.03	21.66
BAI	Male	25.25	27.45	29.69	32.02	33.61
	Female	30.12	33.02	36.38	39.67	42.05
	Total	27.17	30.45	34.15	38.02	40.22
BRI	Male	3.44	4.11	4.87	5,60	6.15
	Female	4.01	4.94	5.88	6.97	7.66
	Total	3.70	4.52	5.49	6.53	7.26
CI	Male	1.21	1.26	1.31	1.35	1.38
	Female	1.24	1.30	1.36	1.43	1.47
	Total	1.22	1.28	1.34	1.40	1.44
WWI	Male	10.30	10.73	11.16	11.59	11.83
	Female	10.88	11.46	12.14	12.80	13.19
	Total	10.55	11.09	11.74	12.43	12.88

Abbreviations: BMI, body mass index; WC, waist circumference; HC, hip circumference; WHtR, waist-to-height ratio; WHR, waist-to-hip ratio; AVI, abdominal volume index; BAI, body adiposity index; BRI, body roundness index; CI, conicity index; WWI, weight-adjusted-waist index.

Table S3 Baseline characteristics between subjects with and without diabetes by sex.

	Male				Female				*P*-value for male vs female
	Total (n=1632)	Diabetes (n=433)	Non-diabetes (n=1199)	*P*-value	Total (n=2220)	Diabetes (n=734)	Non-diabetes (n=1486)	*P*-value	
Age (years)	59.4 ± 14.2	60.2 ± 12.8	59.1 ± 14.7	0.184	63.5 ± 12.8	64.3 ± 11.7	63.1 ± 13.3	0.032*	<0.001***
FPG (mmol/L)	5.34 ± 1.63	6.50 ± 2.67	4.92 ± 0.61	<0.001***	5.52 ± 1.71	6.55 ± 2.57	5.01 ± 0.59	<0.001***	0.001**
TG (mmol/L)	2.20 ± 1.92	2.69 ± 2.30	2.02 ± 1.72	<0.001***	2.07 ± 1.54	2.36 ± 1.91	1.93 ± 1.30	<0.001***	0.666
TC (mmol/L)	4.91 ± 1.26	4.94 ± 1.11	4.89 ± 1.31	0.486	5.18 ± 1.24	5.24 ± 1.22	5.14 ± 1.26	0.076	<0.001***
HDL (mmol/L)	1.24 ± 0.37	1.23 ± 0.46	1.25 ± 0.34	0.261	1.38 ± 0.33	1.37 ± 0.35	1.38 ± 0.32	0.617	<0.001***
LDL (mmol/L)	2.83 ± 0.81	2.84 ± 0.86	2.83 ± 0.79	0.881	2.92 ± 0.79	2.91 ± 0.81	2.92 ± 0.78	0.781	0.001**
UA (μmol/L)	417.20 ± 104.29	415.72 ± 106.12	417.74 ± 103.66	0.733	355.86 ± 96.07	363.30 ± 97.55	352.20 ± 95.16	0.011*	<0.001***
Scr (μmol/L)	86.00 (75.00-99.00)	87.00 (74.50-102.00)	86.00 (75.00-98.00)	0.293	65.00 (57.22-74.00)	65.00 (57.00-76.00)	65.00 (58.00-74.00)	0.235	<0.001***
eGFR (mL/(min·1.73 m²))	83.90 ± 27.05	81.64 ± 25.16	84.72 ± 27.67	0.044*	83.41 ± 21.04	81.96 ± 20.65	84.12 ± 21.20	0.024*	0.528
Weight (kg)	68.29 ± 12.17	70.20 ± 11.85	67.60 ± 12.21	<0.001***	58.47 ± 10.94	60.92 ± 10.76	57.26 ± 10.83	<0.001***	<0.001***
BMI (kg/m²)	25.36 ± 3.81	25.99 ± 3.60	25.13 ± 3.86	<0.001***	25.38 ± 4.04	26.30 ± 4.09	24.93 ± 3.93	<0.001***	0.837
WC (cm)	88.34 ± 9.83	90.46 ± 9.42	87.57 ± 9.87	<0.001***	87.63 ± 9.82	90.09 ± 9.89	86.42 ± 9.56	<0.001***	0.029
WHtR	0.54 ± 0.06	0.55 ± 0.05	0.53 ± 0.06	<0.001***	0.58 ± 0.07	0.59 ± 0.07	0.57 ± 0.07	<0.001***	<0.001***
WHR	0.92 ± 0.06	0.94 ± 0.07	0.92 ± 0.06	<0.001***	0.92 ± 0.06	0.93 ± 0.06	0.91 ± 0.06	<0.001***	<0.001***
AVI	15.86 ± 3.53	16.60 ± 3.51	15.59 ± 3.50	<0.001***	15.62 ± 3.45	16.49 ± 3.57	15.19 ± 3.30	<0.001***	0.036*
BAI	27.55 ± 3.85	28.02 ± 3.77	27.39 ± 3.87	0.004**	33.37 ± 4.93	33.97 ± 5.09	33.08 ± 4.82	<0.001***	<0.001***
BRI	4.19 ± 1.19	4.43 ± 1.13	4.10 ± 1.19	<0.001***	5.05 ± 1.49	5.37 ± 1.55	4.89 ± 1.43	<0.001***	<0.001***
CI	1.26 ± 0.08	1.27 ± 0.08	1.25 ± 0.08	<0.001***	1.30 ± 0.10	1.31 ± 0.09	1.30 ± 0.10	<0.001***	<0.001***
WWI	10.73 ± 0.68	10.83 ± 0.65	10.69 ± 0.69	<0.001***	11.53 ± 0.95	11.60 ± 0.90	11.49 ± 0.98	0.017*	<0.001***

SBP (mmHg)	158.96 ± 22.17	159.19 ± 23.05	158.88 ± 21.85	0.804	161.46 ± 22.93	163.06 ± 23.30	160.67 ± 22.72	0.021*	<0.001***
DBP (mmHg)	95.38 ± 11.88	95.03 ± 12.52	95.51 ± 11.64	0.470	93.93 ± 11.28	93.55 ± 11.20	94.11 ± 11.32	0.275	<0.001***
Smoking (n (%))	734 (44.98%)	189 (43.65%)	545 (45.45%)	0.517	9 (0.41%)	1 (0.14%)	8 (0.54%)	0.161	<0.001***
Drinking (n (%))	193 (11.83%)	48 (11.09%)	145 (12.09%)	0.578	6 (0.27%)	1 (0.14%)	5 (0.34%)	0.393	<0.001***
Family history of diabetes (n (%))	38 (2.38%)	23 (5.35%)	15 (1.29%)	<0.001***	53 (2.40%)	32 (4.38%)	21 (1.42%)	<0.001***	0.969

Continuous data are shown as the mean ± SD or median (Q1-Q3), and categorical data as n (%).

Abbreviations: FPG, fasting plasma glucose; TG, triglycerides; TC, total cholesterol; HDL, high-density lipoprotein; LDL, low-density lipoprotein; UA, urid acid; Scr, serum creatinine; eGFR, estimated glomerular filtration rate; BMI, body mass index; WC, waist circumference; WHtR, waist-to-height ratio; WHR, waist-to-hip ratio; AVI, abdominal volume index; BAI, body adiposity index; BRI, body roundness index; CI, conicity index; WWI, weight-adjusted-waist index; SBP, systolic blood pressure; DBP, diastolic blood pressure.

* *P*-value < 0.05; ** *P*-value < 0.01; *** *P*-value < 0.001.

Table S4 Univariate cox regression models evaluating the association of demographic, biochemical and clinical characteristics, and anthropometric indexes with diabetes.

	HR, 95% CI	*P*-value
Age (years)	1.01 (1.00, 1.01)	0.008**
<44	1.0	-
45-59	1.39 (1.10, 1.75)	0.006**
60-74	1.68 (1.34, 2.10)	<0.001***
≥75	1.38 (1.09, 1.76)	0.009**
Sex (n (%))		
Male	1.0	
Female	1.27 (1.12, 1.43)	<0.001***
TG (mmol/L)	1.10 (1.08, 1.13)	<0.001***
<2.3	1.0	
≥2.3	1.58 (1.40, 1.78)	<0.001***
TC (mmol/L)	1.04 (1.01, 1.08)	0.025*
<6.2	1.0	
≥6.2	1.27 (1.08, 1.49)	0.004**
HDL (mmol/L)	0.98 (0.83, 1.16)	0.812
≥1.0	1.0	
<1.0	1.08 (0.91, 1.28)	0.374
LDL (mmol/L)	1.00 (0.93, 1.07)	0.921
<4.1	1.0	
≥4.1	1.21 (0.97, 1.51)	0.092
UA (μmol/L)	1.00 (1.00, 1.00)	0.768
≤420	1.0	
>420	1.06 (0.94, 1.20)	0.326
Scr (μmol/L)	1.00 (1.00, 1.00)	0.554
eGFR (mL/ (min·1.73 m^2))	1.00 (0.99, 1.00)	0.006**
≥90	1.0	
60-90	1.00 (0.88, 1.13)	0.966
<60	1.25 (1.05, 1.49)	0.012*
Weight (kg)	1.01 (1.01, 1.02)	<0.001***
<75 in male or <65 in female	1.0	
≥75 in male or ≥65 in female	1.40 (1.24, 1.59)	<0.001***
BMI (kg/m^2)	1.06 (1.04, 1.07)	<0.001***
<24.0	1.0	
24.0-28.0	1.40 (1.22, 1.61)	<0.001***
≥28.0	1.80 (1.54, 2.09)	<0.001***
WC (cm)	1.03 (1.02, 1.03)	<0.001***
<90 in male or <80 in female	1.0	
≥90 in male or ≥80 in female	1.64 (1.44, 1.88)	<0.001***
WHtR	49.51 (21.49, 114.04)	<0.001***
<0.5	1.0	
≥0.5	1.80 (1.48, 2.19)	<0.001***
WHR	16.69 (7.52, 37.06)	<0.001***
<0.90 in male or <0.85 in female	1.0	
≥0.90 in male or ≥0.85 in female	1.61 (1.37, 1.90)	<0.001***
AVI	1.07 (1.06, 1.09)	<0.001***
<18	1.0	
≥18	1.62 (1.43, 1.83)	<0.001***
BAI	1.03 (1.02, 1.04)	<0.001***

<34	1.0	
≥34	1.40 (1.24, 1.59)	<0.001***
BRI	1.18 (1.14, 1.23)	<0.001***
<5.5	1.0	
≥5.5	1.54 (1.37, 1.75)	<0.001***
CI	5.79 (3.17, 10.59)	<0.001***
<1.35	1.0	
≥1.35	1.33 (1.17, 1.52)	<0.001***
WWI	1.16 (1.10, 1.23)	<0.001***
<11.5	1.0	
≥11.5	1.35 (1.20, 1.52)	<0.001***
SBP (mmHg)	1.00 (1.00, 1.00)	0.119
<140	1.0	
≥140	0.83 (0.70, 0.98)	0.030*
DBP (mmHg)	0.99 (0.99, 1.00)	0.053
<90	1.0	
≥90	0.93 (0.80, 1.07)	0.302
Smoking (n (%))		
No	1.0	
Yes	0.79 (0.68, 0.93)	0.004**
Drinking (n (%))		
No	1.0	
Yes	0.78 (0.59, 1.04)	0.097
Family history of diabetes (n (%))		
Without DM family history	1.0	
With DM family history	2.38 (1.82, 3.12)	<0.001***

Abbreviations: TG, triglycerides; TC, total cholesterol; HDL, high-density lipoprotein; LDL, low-density lipoprotein; UA, urid acid; Scr, serum creatinine; eGFR, estimated glomerular filtration rate; BMI, body mass index; WC, waist circumference; HC, hip circumference; WHtR, waist-to-height ratio; WHR, waist-to-hip ratio; AVI, abdominal volume index; BAI, body adiposity index; BRI, body roundness index; CI, conicity index; WWI, weight-adjusted-waist index; SBP, systolic blood pressure; DBP, diastolic blood pressure.

* *P*-value < 0.05; ** *P*-value < 0.01; *** *P*-value < 0.001.

Table S5 Multivariate cox regression models evaluating the associations of baseline novel anthropometric indexes with the development of diabetes.

	Unadjusted model		Model 1		Model 2	
	**HR (95%CI)**	***P*-value**	**HR (95%CI)**	***P*-value**	**HR (95%CI)**	***P*-value**
**AVI**						
As continuous variables (per SD increment)	1.31 (1.25, 1.38)	<0.001***	1.34 (1.27, 1.41)	<0.001***	1.31 (1.23, 1.38)	<0.001***
<18	1.0		1.0		1.0	
≥18	1.62 (1.43, 1.83)	<0.001***	1.63 (1.44, 1.84)	<0.001***	1.53 (1.34, 1.73)	<0.001***
**BAI**						
As continuous variables (per SD increment)	1.21 (1.14, 1.28)	<0.001***	1.19 (1.11, 1.27)	<0.001***	1.15 (1.07, 1.23)	<0.001***
<34	1.0		1.0		1.0	
≥34	1.40 (1.24, 1.59)	<0.001***	1.31 (1.14, 1.51)	<0.001***	1.25 (1.08, 1.44)	0.002**
**BRI**						
As continuous variables (per SD increment)	1.31 (1.24, 1.38)	<0.001***	1.29 (1.22, 1.36)	<0.001***	1.26 (1.19, 1.33)	<0.001***
<5.5	1.0		1.0		1.0	
≥5.5	1.54 (1.37, 1.75)	<0.001***	1.46 (1.28, 1.66)	<0.001***	1.38 (1.21, 1.58)	<0.001***
**CI**						
As continuous variables (per SD increment)	1.19 (1.13, 1.26)	<0.001***	1.17 (1.10, 1.24)	<0.001***	1.14 (1.07, 1.22)	<0.001***
<1.35	1.0		1.0		1.0	
≥1.35	1.33 (1.17, 1.52)	<0.001***	1.24 (1.08, 1.43)	0.002**	1.21 (1.04, 1.40)	0.011*
**WWI**						
As continuous variables (per SD increment)	1.17 (1.11, 1.23)	<0.001***	1.13 (1.06, 1.21)	<0.001***	1.11 (1.04, 1.19)	0.003**
<11.5	1.0		1.0		1.0	
≥11.5	1.35 (1.20, 1.52)	<0.001***	1.26 (1.10, 1.45)	<0.001***	1.25 (1.08, 1.44)	0.002**

Abbreviations: AVI, abdominal volume index; BAI, body adiposity index; BRI, body roundness index; CI, conicity index; WWI, weight-adjusted-waist index.

Model 1: adjusted by sex, age, smoking status, drinking status, and family history of diabetes at baseline.

Model 2: adjusted by model 1 plus differences of FPG, TG, TC, HDL, LDL, SBP, DBP between the baseline and follow-up.

* *P*-value < 0.05; ** *P* -value < 0.01; *** *P* -value < 0.001.

Table S6 Multivariate cox regression models evaluating the associations of dynamic changes of anthropometric indices with the development of diabetes.

	Unadjusted model		Model 1		Model 2	
	HR (95%CI)	*P*-value	HR (95%CI)	*P*-value	HR (95%CI)	*P*-value
**Dynamic changes of AVI**						
<18.0 at baseline and follow-up	1.0		1.0		1.0	
<18.0 at baseline and ≥18.0 at follow-up	1.48 (1.20, 1.83)	<0.001***	1.49 (1.21, 1.83)	<0.001***	1.50 (1.21, 1.85)	<0.001***
≥18 at baseline and <18.0 at follow-up	1.52 (1.26, 1.83)	<0.001***	1.51 (1.25, 1.82)	<0.001***	1.46 (1.21, 1.76)	<0.001***
≥18.0 at both baseline and follow-up	1.75 (1.50, 2.03)	<0.001***	1.76 (1.52, 2.05)	<0.001***	1.69 (1.45, 1.97)	<0.001***
**Dynamic changes of BAI**						
<34.0 at baseline and follow-up	1.0		1.0		1.0	
<34.0 at baseline and ≥34.0 at follow-up	1.26 (0.99, 1.60)	0.062	1.20 (0.93, 1.53)	0.162	1.19 (0.92, 1.53)	0.186
≥34.0 at baseline and <34.0 at follow-up	1.32 (1.10, 1.58)	0.003**	1.25 (1.03, 1.51)	0.025*	1.24 (1.02, 1.50)	0.033*
≥34.0 at both baseline and follow-up	1.43 (1.22, 1.66)	<0.001***	1.35 (1.14, 1.60)	<0.001***	1.32 (1.11, 1.57)	0.002***
**Dynamic changes of BRI**						
<5.5 at baseline and follow-up	1.0		1.0		1.0	
<5.5 at baseline and ≥5.5 at follow-up	1.31 (1.05, 1.64)	0.016*	1.24 (0.99, 1.55)	0.061	1.23 (0.98, 1.54)	0.075
≥5.5 at baseline and <5.5 at follow-up	1.35 (1.10, 1.65)	0.004**	1.28 (1.04, 1.58)	0.018*	1.25 (1.01, 1.54)	0.036*
≥5.5 at both baseline and follow-up	1.65 (1.43, 1.91)	<0.001***	1.57 (1.35, 1.82)	<0.001***	1.52 (1.30, 1.77)	<0.001***
**Dynamic changes of CI**						
<1.35 at baseline and follow-up	1.0		1.0		1.0	
<1.35 at baseline and ≥1.35 at follow-up	1.11 (0.91, 1.36)	0.291	1.06 (0.86, 1.30)	0.600	1.03 (0.84, 1.27)	0.753
≥1.35 at baseline and <1.34 at follow-up	1.32 (1.11, 1.56)	0.002**	1.24 (1.04, 1.48)	0.019*	1.20 (1.00, 1.44)	0.048*
≥1.35 at both baseline and follow-up	1.35 (1.12, 1.62)	0.001**	1.25 (1.02, 1.53)	0.028*	1.22 (1.00, 1.50)	0.055
**Dynamic changes of WWI**						
<11.5 at baseline and follow-up	1.0		1.0		1.0	
<11.5 at baseline and ≥11.5 at follow-up	1.16 (0.95, 1.40)	0.138	1.11 (0.91, 1.36)	0.321	1.08 (0.88, 1.33)	0.445
≥11.5 at baseline and <11.5 at follow-up	1.39 (1.17, 1.64)	<0.001***	1.33 (1.11, 1.60)	0.002**	1.30 (1.08, 1.56)	0.005**
≥11.5 at both baseline and follow-up	1.37 (1.18, 1.58)	<0.001***	1.29 (1.08, 1.54)	0.005**	1.26 (1.05, 1.50)	0.013*

Abbreviations: AVI, abdominal volume index; BAI, body adiposity index; BRI, body roundness index; CI, conicity index; WWI, weight-adjusted-waist index.

Model 1: adjusted by sex, age, smoking status, drinking status, and family history of diabetes at baseline.

Model 2: adjusted by model 1 plus differences of FPG, TG, TC, HDL, LDL, SBP, DBP between the baseline and follow-up.

* *P*-value < 0.05; ** *P* -value < 0.01; *** *P* -value < 0.001.

Table S7 Association between WHtR and diabetes according to baseline characteristics.

Subgroup	Diabetes		Non-diabetes		HR (95%CI)	*P*-value	*P*-value for interaction
	WHtR		WHtR				
	<0.5	≥0.5	<0.5	≥0.5			
Age (years)							0.624
<44	9	82	66	265	6.62 (0.06, 720.19)	0.430	
45-59	32	304	149	674	5.82 (0.46, 73.26)	0.173	
60-74	45	464	165	798	1.63 (0.28, 9.60)	0.590	
≥75	24	202	89	465	3.35 (0.31, 35.87)	0.317	
Sex (n (%))							0.563
Male	57	374	290	902	2.60 (0.22, 31.32)	0.452	
Female	53	678	179	1300	2.80 (0.73, 10.71)	0.133	
FPG (mmol/L)							0.005**
<7.0	85	765	465	2187	3.45 (0.88, 13.54)	0.076	
≥7.0	23	282	0	1	1.00 (0.11, 9.17)	1.000	
TG (mmol/L)							0.008**
<2.3	89	632	423	1584	3.03 (0.71, 12.82)	0.133	
≥2.3	20	412	42	604	2.38 (0.34, 16.86)	0.384	
TC (mmol/L)							0.248
<6.2	98	877	428	1907	3.77 (1.05, 13.47)	0.041	
≥6.2	11	168	37	281	0.76 (0.05, 12.40)	0.845	
HDL (mmol/L)							0.872
≥1.0	101	902	427	1902	2.95 (0.88, 9.88)	0.079	
<1.0	8	143	38	283	1.81 (0.03, 98.53)	0.771	

LDL (mmol/L)							0.906
<4.1	105	959	451	2040	3.34 (0.99, 11.22)	0.051	
≥4.1	4	81	11	143	0.47 (0.01, 32.72)	0.727	
UA (μmol/L)							0.827
≤420	85	657	335	1426	3.83 (0.90, 16.18)	0.068	
>420	20	369	115	730	1.28 (0.17, 9.89)	0.814	
eGFR (mL/(min·1.73 m²))							0.169
≥90	35	402	193	862	2.88 (0.18, 47.24)	0.459	
60-90	53	479	215	1056	2.14 (0.38, 11.95)	0.386	
<60	19	155	55	258	5.16 (0.70, 38.15)	0.108	
Weight (kg)							0.867
<56.50	68	249	300	651	4.17 (0.29, 59.66)	0.293	
56.50-67.82	29	365	123	755	3.27 (0.31, 34.97)	0.326	
≥67.82	13	438	46	796	2.25 (0.28, 18.37)	0.448	
BMI (kg/m²)							0.776
≤24.0	94	240	421	669	5.90 (0.18, 193.71)	0.319	
24.0-28.0	15	482	47	1024	2.59 (0.22, 30.60)	0.450	
≥28.0	1	330	1	509	4.03 (0.35, 45.94)	0.261	
WC (cm)							0.380
<90 in male or <80 in female	107	192	467	580	2.04 (0.01, 386.16)	0.790	
≥90 in male or ≥80 in female	3	860	2	1622	2.83 (0.72, 11.20)	0.138	
WHR							0.117
<0.90 in male or <0.85 in female	66	99	334	280	5.35 (0.04, 817.09)	0.513	
≥0.90 in male or ≥0.85 in female	44	953	134	1921	2.94 (0.87, 9.92)	0.082	
AVI							0.189
<18	110	679	468	1664	2.17 (0.30, 15.74)	0.444	

≥18	0	373	0	537	3.08 (0.42, 22.43)	0.266	
BAI							0.103
<34	109	685	468	1589	3.48 (0.55, 22.14)	0.187	
≥34	1	367	0	612	2.41 (0.35, 16.44)	0.368	
BRI							0.097
<5.5	110	673	469	1627	1.70 (0.15, 19.82)	0.673	
≥5.5	0	379	0	575	1.98 (0.18, 21.91)	0.576	
CI							0.020*
<1.35	109	752	469	1683	3.44 (0.68, 17.40)	0.136	
≥1.35	1	300	0	519	2.38 (0.33, 17.00)	0.388	
WWI							0.166
<11.5	108	601	459	1405	2.24 (0.29, 17.03)	0.437	
≥11.5	2	451	10	797	2.88 (0.56, 14.72)	0.203	
DBP							0.830
<90	82	844	373	1774	3.74 (0.22, 63.13)	0.360	
≥90	28	208	96	428	3.16 (0.89, 11.19)	0.074	
Smoking (n (%))							0.405
No	84	888	329	1790	3.04 (0.89, 10.39)	0.077	
Yes	26	164	140	412	2.67 (0.08, 88.34)	0.583	
Drinking (n (%))							0.838
No	108	1005	435	2086	3.01 (0.92, 9.80)	0.068	
Yes	2	47	34	116	4.13 (0.02, 1018.99)	0.614	
DM family history (n (%))							0.777
Without DM family history	106	995	450	2147	3.29 (1.03, 10.58)	0.045	
With DM family history	3	52	8	28	0.91 (0.00, 1736.51)	0.980	

Abbreviations: FPG, fasting plasma glucose; TG, triglycerides; TC, total cholesterol; HDL, high-density lipoprotein; LDL, low-density lipoprotein; UA, urid acid; Scr, serum creatinine; eGFR, estimated glomerular filtration rate; BMI, body mass index; WC, waist circumference; WHtR, waist-to-height ratio; WHR, waist-to-hip ratio; AVI, abdominal volume index; BAI, body adiposity index; BRI, body roundness index; CI, conicity index; WWI, weight-adjusted-waist index; SBP, systolic blood pressure; DBP, diastolic blood pressure.

[*] *P*-value < 0.05; [**] *P*-value < 0.01; [***] *P*-value < 0.001.
